# Diagnosed Incidence of Non-Affective Psychotic Disorders Amongst Adolescents in British Columbia and Sociodemographic Risk Factors: A Retrospective Cohort Study

**DOI:** 10.1177/07067437211055412

**Published:** 2021-11-18

**Authors:** Carly Magee, Martin Guhn, Joseph H. Puyat, Anne Gadermann, Eva Oberle

**Affiliations:** 1 School of Population and Public Health (SPPH), 8166 University of British Columbia, Vancouver, British Columbia, Canada; 2 Human Early Learning Partnership (HELP), 8166 University of British Columbia, Vancouver, British Columbia, Canada; 3 Centre for Health Evaluation and Outcome Sciences (CHEOS), St. Paul’s Hospital, Vancouver, British Columbia, Canada

**Keywords:** adolescence, early-onset schizophrenia, immigrant mental health, psychosis, refugee, schizophrenia, sex, income inequality, health disparities

## Abstract

**Objectives:**

To estimate the diagnosed incidence of non-affective psychotic disorder between the ages of 13 and 19 years in South-Western British Columbia (BC) and to examine variation in risk by sex, family and neighbourhood income, family migration background, parent mental health contact and birth year.

**Methods:**

Linked individual-level administrative data were used to construct a cohort of individuals born in 1990–1998 and residing in South-Western BC (*n* = 193,400). Cases were identified by either one hospitalization or two outpatient physician visits within 2 years with a primary diagnosis of a non-affective psychotic disorder (ICD-10: F20–29, ICD-9: 295, 297, 298). We estimated cumulative incidence, annual cumulative incidence and incidence rate between the ages of 13 and 19 years, and conducted Cox proportional hazards regression to estimate associations between sociodemographic factors and risk over the study period.

**Results:**

We found that 0.64% of females and 0.88% of males were diagnosed with a non-affective psychotic disorder between the ages of 13 and 19 years, with increasing risk observed over the age range, especially amongst males. Incidence rate over the entire study period was 106 per 100,000 person-years for females and 145 per 100,000 person-years for males. Risk of diagnosis was elevated amongst those in low-income families and neighbourhoods, those with a parent who had a health service contact for a mental disorder, and more recent birth cohorts. Risk was reduced amongst children of immigrants compared to children of non-migrants.

**Conclusions:**

Findings from this study provide important information for health service planning in South-Western BC. Future work should examine whether variations in diagnosed incidence is driven by differences in health service engagement or reflect genuine differences in risk.

## Abstract (fr)

**Objectifs:**

Estimer l’incidence diagnostiquée des troubles psychotiques non affectifs à l’âge de 13 à 19 ans dans le sud-ouest de la Colombie-Britannique (C.-B.) et examiner la variation des risques selon le sexe, la famille, et le revenu du quartier, les antécédents de la migration familiale, les contacts de santé mentale des parents et l’année de naissance.

**Méthodes:**

Des données administratives couplées au niveau individuel ont été utilisées pour former une cohorte de personnes nées entre 1990 et 1998 et habitant le sud-ouest de la C.-B. (*N* = 193 400). Les cas ont été identifiés soit par une hospitalisation, soit par deux visites ambulatoires à un médecin dans les deux ans précédents, avec un diagnostic primaire de trouble psychotique non affectif (CIM-10 : F20-29, CIM-9 : 295, 297, 298). Nous avons estimé l’incidence cumulative, l’incidence cumulative annuelle et le taux d’incidence entre l’âge de 13 ans et de 19 ans, et mené une régression aléatoire proportionnelle de Cox pour estimer les associations entre les facteurs sociodémographiques et les risques sur la période de l’étude.

**Résultats:**

Nous avons observé que 64% des filles et 88% des garçons ont été diagnostiqués d’un trouble psychotique non affectif entre l’âge de 13 ans et de 19 ans, avec un risque accru observé sur l’intervalle d’âge, spécialement chez les sujets de sexe masculin. Le taux d’incidence sur toute la période de l’étude était de 106 par 100 000 années-personnes pour les filles et de 145 par 100 000 années-personnes pour les garçons. Le risque d’un diagnostic était élevé chez ceux appartenant à des familles et des quartiers à faible revenu, ceux dont un parent avait un contact d’un service de santé pour un trouble mental, et des cohortes de naissance plus récente. Le risque était réduit chez les enfants d’immigrants comparé aux enfants de non-migrants.

**Conclusions:**

Les résultats de cette étude offrent une information importante pour la planification des services du sud-ouest de la C.-B. La future recherche devrait examiner si la variation de l’incidence diagnostiquée est alimentée par les différences d’engagement au service de santé ou si elle reflète de véritables différences des risques.

## Introduction

Schizophrenia and related psychotic disorders are associated with high levels of disability and health service use over the lifespan.^1,2^ Onset during adolescence is common^3,4^ and is associated with greater impairment compared to onset during adulthood.^5,6^ Estimates of the number of adolescents expected to develop psychotic disorders are essential for early psychosis intervention services and education planning.^4,7^ In addition, knowledge of risk factors and changes in incidence over time may inform resource allocation and provide insight into the aetiology of these conditions.

Estimates of the annual incidence of non-affective psychotic disorder amongst adolescents range from approximately 30 to 150 per 100,000.^4,7–10^ Risk increases over the adolescent age range, and cumulative incidence by the end of adolescence has been found to range from 0.04% to 0.8%.^7,9–14^ Incidence estimates are higher in studies with more inclusive case definitions and in studies that identify cases through administrative health data as opposed to entry into specialized health programs.^4^

Risk of adolescent-onset psychotic disorders appears to be greater amongst males compared to females, although this sex difference does not typically emerge until mid to late adolescence^8,9,12,13^ and has been found in some studies of early-onset cases to be reversed.^10^ Moreover, numerous studies have found that the risk of psychotic disorders is elevated amongst children of parents with mental disorders^15^ and amongst a number of socially marginalized groups, including those who are from low-income families, living in disadvantaged areas, ethnic minorities and certain immigrant groups.^8,16–21^ Finally, numerous studies have found evidence of increasing incidence of psychotic disorders amongst young people in particular.^9,12,22,23^ For example, a Danish study found that annual incidence of schizophrenia amongst individuals aged 12–18 was three times greater over the period 1994–2010 compared to 1971–1993.^23^ A number of studies have found that increasing incidence is most pronounced for psychotic disorders other than schizophrenia and for cases identified through outpatient as opposed to inpatient settings.^12,22^

Further research with recent population samples is needed to better understand the number of adolescents affected by schizophrenia and related psychotic disorders, associated risk factors and changes in incidence over time. Research in local contexts is needed as meta-analyses have found substantial variation in incidence between regions^24^ and limited research has been conducted in British Columbia (BC).^9^ In the current study, our objectives were to (1) estimate diagnosed incidence of non-affective psychotic disorder between the ages of 13 and 19 years in South-Western BC and (2) examine variation in risk by several sociodemographic factors (e.g., sex, low family income, neighbourhood income, family migration background, parent mental health contact history and birth year).

We expected to find estimates similar to recent studies based on administrative health data and to find higher diagnosed incidence amongst males, individuals from low-income families and neighbourhoods, individuals with parents with mental health contact history and amongst those born more recently. The question of whether family migration background was related to risk was exploratory. Meta-analyses indicate that the first- and second-generation immigrants have two to three times the risk of psychotic disorders compared to the general population.^17–20^ However, a Canadian study^16^ found that while certain groups exhibited elevated risk (i.e., refugees and immigrants from the Caribbean and Bermuda), first-generation immigrants as a group had a similar level of risk as to the general population. We are not aware of any Canadian studies that have examined associations between family migration background and incidence over adolescence.

## Methods

### Study Design and Cohort

This was a retrospective cohort study of incidence and variation in risk of diagnosed non-affective psychotic disorder between the ages of 13 and 19 years. We examined incidence starting at the age of 13 years as this is the minimum age eligible for early psychosis intervention services and because the validity of psychosis diagnoses in health data for younger children is not well understood.^9^ This study was conducted as part of a larger project examining health, well-being and academic outcomes of children of migrants compared to children of non-migrants in the 10 largest school districts in BC. These districts are all located in South-Western BC and capture 78% of the immigrant population in the province.^25^ The study cohort included all individuals born between January 1990 and March 1998 who attended public school in these districts and were enrolled in the provincial health plan (covers 99% of the population)^9^ at the age of 13 years (*n* = 216,173).

Individuals were followed from their 13th birthday until the date of the first diagnosis for a non-affective psychotic disorder, the end of their enrolment in the provincial health plan (determined on an annual basis), or their 19th birthday, whichever event came first. This observation period corresponds to calendar years 2003– 2017, depending on individuals’ birth year. To be included in the study, individuals were required to have been enrolled in the provincial health plan for a clearance period of 3 years prior to study inception (*n* = 193,495). Those diagnosed with a non-affective psychotic disorder during this period were excluded as prevalent cases (*n* = 92).

### Data Sources

Data sources included records of school registration, outpatient physician visits, hospitalizations, registration in the provincial health plan and linked postal code and census information for the study cohort.^26–29^ In addition, the study used records of outpatient physician visits and records of immigration to Canada for parents of the study cohort.^30,31^ Individual-level data were linked by Population Data BC with a probabilistic–deterministic approach using multiple personal identifiers (linkage rate for study cohort = 98.4%; linkage rate for parents to study cohort = 95.0%). This study was approved by the University of British Columbia’s Behavioural Research Ethics Board (UBC BREB: H10-01154).

### Case Ascertainment

Cases were defined as individuals who received a primary diagnosis of a non-affective psychotic disorder in at least one hospitalization (ICD-10: F20–29) or at least two outpatient physician visits within 2 years (ICD-9: 295, 297, 298). Diagnoses of substance-induced psychosis were not included. We created this algorithm based on existing algorithms from Canadian and International research.^4,10,32,33^ It is similar to one validated for detecting chronic psychotic illness in Ontario^33^ (ICD-10: F20, F25, F29; ICD-9: 295, 298), but includes the full range of non-affective psychotic disorder diagnoses as our focus was on the first episodes as opposed to existing chronic illness.

### Explanatory Variables

Sex (female, male) and birth year were taken from records of registration in the provincial health plan.^29^ Birth years were categorized into three groups (i.e., 1990–1992, 1993–1995, 1996–1998) on the basis of similar levels of risk. An individuals’ family receiving a full premium subsidy for the provincial health plan at any time when they were aged 10–13 was used as a proxy for low family income at the start of the study period (yes, no).^27^ Neighbourhood income quintile at the age of 13 years (ranged from 1 to 5, with 1 representing the lowest quintile) was taken from the consolidation files that link postal code to neighbourhood income data from the most recent census.^29^

Family migration background (e.g., child of non-migrant, immigrant, refugee) was determined based on one or two parents having a record in Immigration Refugees and Citizenship Canada’s Permanent Resident Database.^30^ Individuals with one immigrant and one refugee parent were classified as a child of a refugee. Children of migrants included both first- and second-generation migrants. Note that immigration data only extended back to 1985; as such, children of parents who migrated prior to this year were included in the reference group. Recent parent mental health contact^31^ at the start of the study reflected whether either of the child’s parents had an outpatient contact with a primary diagnosis for a non-affective psychotic disorder (ICD-9: 295, 297, 298), an anxiety, mood or substance use disorder (ICD-9: 50B, 291, 292, 296, 300, 303–305, 311)^34,35^ or no mental disorder in the years when the individual was at the ages of 10–13 years. We restricted the observation of parent mental health contact to this period to ensure approximately equal opportunity to observe mental health contacts amongst parents.

### Analyses

Descriptive statistics were calculated for all study variables. Kaplan–Meier survival analysis was used to estimate the probability of ever receiving a new diagnosis for a non-affective psychotic disorder between the ages of 13 and 19 years (‘cumulative incidence’) as well as the probability of new diagnosis within each year of life between the ages of 13 and 19 years (‘annual cumulative incidence’). In addition, we calculated the incidence rate per 100,000 person-years over the entire study period. All estimates were reported separately by sex and with 95% confidence intervals. These are measures of ‘diagnosed incidence’ as they capture only those cases diagnosed in the health system.^36^

Cox proportional hazards regression with years of follow-up as time was used to examine associations between explanatory variables and risk of diagnosis over the study period.^1^ Due to finding non-proportional hazards by sex, regressions were conducted separately for females and males. Unadjusted and adjusted hazard ratios (aHR) with 95% confidence intervals were reported for all explanatory variables. 95% confidence intervals that did not cross 1.0 were taken to indicate statistical significance at α = 0.05. All analyses were conducted in SAS software release 9.4.^37^

### Missing Data

Missing data on study variables was low (0.85% on neighbourhood income and 2.39% on low family income, and 3.2% on either). Less than five individuals with missing data on sex were excluded from the analyses. Individuals with missing data on neighbourhood income and/or low family income were retained and ‘missing’ was coded as a level of each variable.

### Sensitivity Analyses

We examined whether any change in risk over birth cohorts was observed when we included only cases diagnosed in hospital and only cases diagnosed with schizophrenia specifically (ICD-10 F20; ICD-9 295). In addition, we reran the analyses using a validated algorithm^33^ to detect chronic psychotic illness to examine the extent to which findings were sensitive to differences between the algorithms. To assess the potential for missing data to bias the results, we conducted sensitivity analyses assuming specific values for missing data (low family income = 0 or 1 and neighbourhood income quintile = 1 or 5).

## Results

### Descriptive Statistics

Table 1 shows the descriptive statistics. Individuals were followed for an average of 5.79 years and a total of 1,117,852 person-years. Nearly three quarters of the cohort were children of non-migrants, almost one quarter were children of immigrants and less than 4% were children of refugees. Close to one-third of the sample had low family income between the ages of 10 and 13 years and the sample was approximately evenly distributed across neighbourhood income quintiles. Approximately 40% of the sample had a parent with a mental health contact, and 1% had a parent with a contact for a non-affective psychotic condition, in the years when they were at the ages of 10–13 years. An equal number of individuals were born between 1990 and 1992 and 1993 and 1995 and fewer were born between 1996 and 1998 due to the fact that the 1998 birth cohort only included individuals born between January and March of that year.

**Table 1.** Descriptive Statistics.

	Females (*n* = 93,784)			Males (*n* = 99,616)		
	*n* (% sample)	*n* cases	Person-years	*n* (% sample)	*n* cases	Person-years
Low family income						
No	63,770 (68.00)	308	372,416.80	67,891 (68.15)	473	396,483.44
Yes	27,748 (29.59)	263	157,525.40	29,357 (29.47)	346	166,747.76
Missing	2,266 (2.42)	5	11,715.22	2,368 (2.38)	14	12,337.28
Neighbourhood income quintile						
5 (highest)	16,798 (17.91)	92	97,764.36	17,847 (17.92)	112	103,869.54
4	18,997 (20.26)	87	110,372.57	20,280 (20.36)	156	118,029.60
3	19,603 (20.90)	122	113,305.34	20,538 (20.62)	159	118,915.02
2	19,542 (20.84)	121	112,561.92	20,884 (20.96)	201	120,500.68
1 (lowest)	18,024 (19.22)	149	102,917.04	19,243 (19.32)	190	109,877.53
Missing	820 (0.87)	5	4,649.40	824 (0.83)	15	4,663.84
Family migration background						
Non-migrant	68,977 (73.55)	453	399,169.90	72,198 (72.48)	657	418,026.42
Immigrant	21,579 (23.01)	102	124,295.04	24,082 (24.17)	151	138,953.14
Refugee	3,228 (3.44)	21	18,367.32	3,336 (3.35)	25	18,981.84
Parent MH contact						
None	56,415 (60.15)	263	326,642.85	59,543 (59.77)	373	344,753.97
Anxiety/mood/substance	36,360 (38.77)	290	209,397.24	38,976 (39.13)	425	224,501.76
Non-affective psychotic	1,009 (1.08)	23	5,669.57	1,097 (1.10)	35	6,165.14
Birth year						
1990–1992	34,776 (37.06)	172	199,847.00	36,515 (36.66)	259	209,596.10
1993–1995	34,398 (36.68)	224	198,820.44	36,955 (37.10)	321	213,599.90
1996–1998	24,630 (26.26)	180	142,854.00	26,146 (26.25)	253	152,169.72
Total	93,784 (100.00)	576	542,071.52	99,616 (100.00)	833	575,780.48

**Table 2.** Annual Cumulative Incidence Within Each Year of Life Between the Ages of 13 and 19 Years.

Year of life	Females (*n* = 93,784)		Males (*n* = 99,616)	
	# First diagnoses	Annual cumulative incidence per 100,000 [95% CI]	# First diagnoses	Annual cumulative incidence per 100,000 [95% CI]
13–14	40	43 [31, 58]	46	46 [35, 62]
14–15	75	82 [65, 102]	73	75 [59, 94]
15–16	100	110 [90, 133]	137	141 [119, 167]
16–17	124	137 [115, 163]	140	146 [123, 172]
17–18	111	124 [103, 149]	186	196 [169, 226]
18–19	126	147 [124, 175]	251	274 [242, 310]

*Note*: Annual cumulative incidence of each year corresponds to the probability of new diagnosis within each 1-year period.

### Diagnosed Incidence

We identified 1,409 cases (576 female and 833 male) of non-affective psychotic disorder onset between the ages of 13 and 19 years. The median age of onset was 17 for both males and females. Approximately three quarters (76%) of the first diagnoses were in outpatient as opposed to inpatient settings, and the majority were for schizophrenia (ICD-9 295 and ICD-10 F20: 46%) and unspecified/other psychotic disorders (ICD-9 298 and ICD-10 F29: 46%).

**Figure 1.**
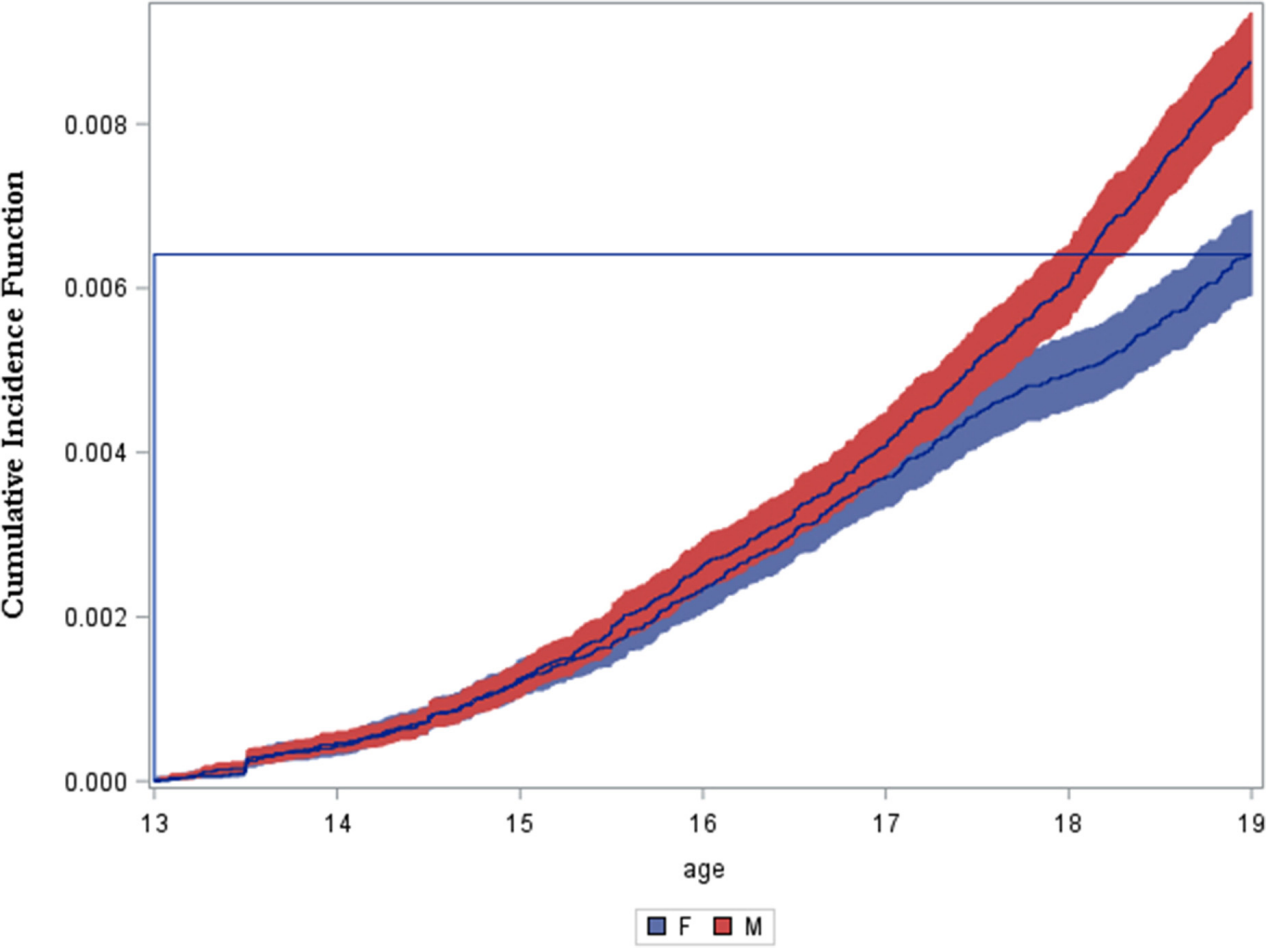
Cumulative incidence function between the ages of 13 and 19 years by sex.

Cumulative incidence of non-affective psychotic disorder by the age of 19 years was 0.64% [95% CI: 0.59 to 0.70] amongst females and 0.88% [95% CI: 0.82 to 0.94] amongst males. Higher cumulative incidence amongst males compared to females emerged after the age of 15 years and this sex difference became more pronounced between the ages of 17 and 19 years (Figure 1). Annual cumulative incidence within each year of life ranged from 43 to 147 per 100,000 for females and 46 to 274 per 100,000 for males, with a steeper increase in incidence observed for males (Table 2). Incidence rate over the entire study period was 106 per 100,000 person-years [95% CI: 98 to 115] for females and 145 per 100,000 person-years [95% CI: 135 to 155] for males.

**Table 3.** Cox Proportional Hazards Results.

	Females (*n* = 93,784)		Males (*n* = 99,616)	
	HR [95% CI]	aHR [95% CI]	HR [95% CI]	aHR [95% CI]
Low family income				
No	Reference	Reference	Reference	Reference
Yes	2.03 [1.72, 2.39]	1.97 [1.66, 2.35]	1.75 [1.53, 2.02]	1.70 [1.47, 1.96]
Missing	0.53 [0.22, 1.28]	0.62 [0.25,1.49]	0.98 [0.58, 1.67]	1.14 [0.67, 1.95]
Neighbourhood income quintile				
5 (highest)	Reference	Reference	Reference	Reference
4	0.84 [0.62, 1.12]	0.81 [0.60,1.08]	1.23 [0.96, 1.56]	1.18 [0.92, 1.50]
3	1.14 [0.87, 1.50]	1.07 [0.81, 1.40]	1.24 [0.98, 1.58]	1.17 [0.92, 1.50]
2	1.14 [0.87, 1.50]	1.00 [0.76, 1.32]	1.55 [1.23, 1.96]	1.42 [1.12, 1.79]
1 (lowest)	1.54 [1.19, 2.00]	1.26 [0.96, 1.64]	1.61 [1.28, 2.04]	1.41 [1.11, 1.79]
Missing	1.15 [0.47, 2.82]	0.90 [0.36, 2.22]	3.01 [1.76, 5.16]	2.46 [1.43, 4.24]
Family migration background				
Non-migrant	Reference	Reference	Reference	Reference
Immigrant	0.73 [0.59, 0.90]	0.67 [0.54, 0.83]	0.69 [0.58, 0.83]	0.65 [0.55, 0.78]
Refugee	1.01 [0.65, 1.57]	0.77 [0.49, 1.20]	0.84 [0.56, 1.25]	0.67 [0.45, 1.01]
Parent MH contact				
None	Reference	Reference	Reference	Reference
Anxiety/mood/substance	1.32 [1.07, 1.62]	1.63 [1.38, 1.93]	1.28 [1.08, 1.52]	1.67 [1.45, 1.92]
Non-affective psychotic	6.09 [3.03, 12.24]	3.90 [2.54, 5.99]	3.00 [1.34, 6.71]	4.35 [3.07, 6.16]
Birth year				
1990–1992	Reference	Reference	Reference	Reference
1993–1995	1.31 [1.07, 1.60]	1.32 [1.08, 1.61]	1.22 [1.03, 1.43]	1.22 [1.03, 1.43]
1996–1998	1.46 [1.18, 1.80]	1.51 [1.22, 1.86]	1.34 [1.13, 1.60]	1.39 [1.17, 1.65]

*Notes*: HR:  unadjusted hazard ratio; aHR: adjusted hazard ratio. aHR is adjusted for all other explanatory variables.

### Cox Proportional Hazards Model Results

Table 3 shows the regression results by sex with 95% confidence intervals. Low family income was associated with significantly elevated risk of diagnosis (aHR females = 1.97[95% CI: 1.66 to 2.35] and aHR males = 1.70[95% CI: 1.47 to 1.96]). Amongst females, the hazard ratio comparing the lowest and highest neighbourhood income quintiles was statistically significant in the unadjusted model only (HR = 1.54[95% CI: 1.19 to 2.00]). Amongst males, the lowest two quintiles (aHR = 1.41[95% CI: 1.11 to 1.79] and aHR = 1.42[95% CI: 1.12 to 1.79]) and those with missing neighbourhood income information (aHR = 2.46[95% CI: 1.43 to 4.24]) exhibited significantly elevated risk compared to the highest quintile. Children of immigrants had significantly lower risk of diagnosis compared to children of non-migrants (aHR females = 0.67[95% CI: 0.54 to 0.83] and aHR males = 0.65[95% CI: 0.55 to 0.78]). Children of refugees had lower risk of diagnosis compared to children of non-migrants (aHR females = 0.77[95% CI: 0.49 to 1.20] and aHR males = 0.67[95% CI: 0.45 to 1.01); however, these effects were not statistically significant in any models. Finally, elevated risk of diagnosis was observed amongst individuals with a parent with a health contact for an anxiety/mood/substance disorder (aHR females = 1.63[95% CI: 1.38 to 1.93] and aHR males = 1.67[95% CI: 1.45 to 1.92]), and for a non-affective psychotic disorder (aHR females = 3.90[95% CI: 2.54 to 5.99] and aHR males = 4.35[95% CI: 3.07 to 6.16]) and amongst those born in 1993–1095 (aHR females = 1.32[95% CI: 1.08 to 1.61] and aHR males = 1.22[95% CI: 1.03 to 1.43]) and 1996–1998 (aHR females = 1.51[95% CI: 1.22 to 1.86] and aHR males = 1.39[95% CI: 1.17 to 1.65]) compared to those born in 1990–1992.

### Sensitivity Analyses Results

When we restricted our case definition to hospital diagnoses, we still observed an increase in risk over birth year categories; however, the magnitude of these effects was reduced and they were no longer statistically significant. Amongst females, aHR = 1.11[95% CI: 0.78 to 1.57] and aHR = 1.24[95% CI: 0.86 to 1.81] and amongst males, aHR = 1.13[95% CI: 0.88 to 1.45] and aHR = 1.24[95% CI: 0.95 to 1.63] for 1993–1995 and 1996–1998, respectively, compared to 1990–1992. In addition, we found that incidence of schizophrenia specifically increased over birth years, although the magnitude of effects was attenuated particularly amongst females. Amongst females, aHR = 1.21[95% CI: 0.92 to 1.59] and aHR = 1.39[95% CI: 1.04 to 1.85] and amongst males, aHR = 1.17[95% CI: 0.96 to 1.43] and aHR = 1.36[95% CI: 1.10 to 1.68] for 1993–1995 and 1996–1998, respectively, compared to 1990–1992.

Using the validated algorithm to detect chronic psychotic illness,^32^ estimated incidence was reduced by 14% amongst males and 23% amongst females (see Supplemental Material). Findings from regression analyses were consistent with the original models with the exception that the comparison between 1993 and 1995 and 1990 and 1992 birth cohorts was no longer statistically significant (Table S1, Supplemental Material). Findings were consistent across models assuming different values for missing data (results are not reported).

## Discussion

### Main Findings and Implications

We found that .64% of females and 0.88% of males were diagnosed with a non-affective psychotic disorder between the ages of 13 and 19 years. This corresponded to an incidence rate of 106 per 100,000 person-years for females and 145 per 100,000 person-years for males over the entire age range. Using a more restrictive algorithm validated to detect chronic psychotic illness^33^ resulted in lower incidence estimates, particularly amongst females. These estimates are similar to other recent studies that have used administrative health data to measure incidence of psychotic disorders over adolescence,^4,7–12^ but are considerably higher than annual treated incidence in early psychosis intervention programs (13–18 per 100,000).^38,39^ Further research should investigate whether adolescents experience barriers to accessing such programs.

Our findings of increasing risk over the adolescent age range and elevated risk amongst males compared to females emerging in mid-adolescence are consistent with prior research,^9,11–14^ as are the findings of elevated risk amongst children of individuals with mental health contact history^15^ and individuals from low SES backgrounds.^4,21^ Few studies include measures of both area and family level disadvantage and our findings indicate that both are associated with added risk. These findings may reflect that growing up in disadvantaged environments increases the likelihood of developing psychotic disorders or that parents with mental illnesses are more likely to both be low income and have children with psychotic disorders. However, we found that the effects of socio-economic disadvantage on risk were observed even while adjusting for parent mental health contacts, similar to findings from a recent study conducted in Denmark.^21^ These findings highlight the importance of poverty reduction and indicate that lower income areas may benefit from a higher concentration of early psychosis intervention services.

The finding of lower risk of diagnosed non-affective psychotic disorder amongst children of immigrants contrasts with international findings that first- and second-generation immigrants exhibit elevated risk,^17–20^ but is similar to findings from Ontario^16^ that first-generation immigrants as a group exhibited non-significant reduced risk (9%) compared to non-immigrants. We found a larger discrepancy between immigrant and non-immigrant groups, potentially due to the focus on children of migrants or to differences in the immigrant populations^25^ (e.g., BC has a larger proportion of immigrants from Europe and East Asia, Groups found to exhibit reduced risk of psychotic disorders in Ontario). Our finding that children of refugees did not exhibit elevated risk of diagnosis was surprising given the social, psychological and economic challenges faced by refugee families^40^ and contrasts with findings from Ontario that first generation refugees exhibited elevated risk.^16^ However, our sample included a relatively small number of children from refugee families.

The contrast between our findings and international research may be due to differences in immigrant populations. The majority of immigrants to Canada are selected on the basis of economic position and many studies have found that they report better health across a number of indicators.^41^ Alternatively, lower rates of diagnosis amongst children of migrants may reflect differences in health service engagement as first- and second-generation immigrants are less likely to access mental health services in Canada.^42^ Future work should assess how the risk of adolescent-onset psychotic conditions may vary amongst children of migrants in BC and whether differences in health service engagement for children of migrants may drive the lower observed risk.

Finally, our finding that diagnosed incidence was higher amongst individuals born later is consistent with a number of recent health registry studies.^9,12,22,23^ This may reflect a genuine increase in incidence, or that these conditions are being detected earlier or more frequently. The latter is likely given the proliferation of early psychosis intervention services in recent decades^43^ and is supported by our finding that increased risk over birth years is largely driven by outpatient diagnoses. These findings highlight the importance of ongoing population surveillance of psychotic disorders.

### Study Strengths and Limitations

The use of administrative health data allowed us to make population-based estimates of incidence of non-affective psychotic disorders over adolescence. A large population sample is essential for studying these rare conditions and enabled us to examine risk of diagnosis by several sociodemographic variables, including measures of both neighbourhood and family level SES, family migration background and parent mental health contact history.

This study was limited by several factors. Our algorithm for detecting cases has not been validated, although a similar algorithm that was validated in Ontario^33^ to detect chronic psychotic illness was found to have high sensitivity (94%) and moderate positive predictive value (62%). In addition, because our algorithm did not include secondary/tertiary diagnoses of psychotic disorders we may have missed cases presenting with multiple conditions. Furthermore, we were unable to identify cases diagnosed in the emergency department but not admitted to the hospital and cases diagnosed outside of the health system, such as those diagnosed by counsellors/social workers in child and youth community mental health services. We note, however, that it is the policy of such services to refer suspected cases of psychosis to specialized health services.^43,44^

This study was also limited by the fact that it excluded adolescents who were not enrolled in public school (about 12% of the population).^45^ Given that higher income families are over-represented in private schools,^46^ estimates of diagnosed incidence in our study are likely elevated compared to the total population. Moreover, we likely missed some individuals from low-income households as our proxy measure relied on low-income families opting into subsidy assistance for health plan premiums.^47^ Similarly, we were unable to identify children of migrants who arrived prior to 1985. As such, effect estimates for low family income and family migration background may be attenuated. Finally, our measures of neighbourhood income, low family income and parent mental health contact were taken at the start and immediately before the observation period and may not reflect individuals’ current socioeconomic position and parent mental health at the time of the first diagnosis.

## Conclusion

Diagnosed incidence of adolescent-onset non-affective psychotic disorders has increased in recent years and the risk of diagnosis varies considerably by sex, family and neighbourhood income, family migration background and parent mental health contact history amongst adolescents in South-Western BC. More research is needed to understand the extent to which variation in diagnosed incidence reflects genuine differences in risk or differential detection rates in the health system, and whether adolescents with psychotic disorders experience barriers to accessing appropriate health services.

## Data Access

Access to the data extract used for this study was granted by Population Data BC, a trusted third-party organization that securely stores data from partner organizations (e.g., Ministry of Health). Access to the data is granted only to approved members of the research team and the extract will be destroyed after the study concludes.

## Supplemental Material

sj-docx-1-cpa-10.1177_07067437211055412 - Supplemental material for Diagnosed Incidence of Non-Affective Psychotic Disorders Amongst Adolescents in British Columbia and Sociodemographic Risk Factors: A Retrospective Cohort Study Supplemental material, sj-docx-1-cpa-10.1177_07067437211055412 for Diagnosed Incidence of Non-Affective Psychotic Disorders Amongst Adolescents in British Columbia and Sociodemographic Risk Factors: A Retrospective Cohort Study by Carly Magee, Martin Guhn, Joseph H. Puyat, Anne Gadermann and Eva Oberle in The Canadian Journal of Psychiatry

## Notes

1. Note that hazard ratios produced by Cox regression were almost identical to risk ratios produced by Poisson regression with the logarithm of person-years as the offset.
