# Supplementary material for: Diagnosed Incidence of Non-Affective Psychotic Disorders Amongst Adolescents in British Columbia and Sociodemographic Risk Factors: A Retrospective Cohort Study
Source: Can J Psychiatry. 2021 Nov 18;67(4):295–304. doi: 10.1177/07067437211055412 (PMC9014684; doi:10.1177/07067437211055412)
Supplement: sj-docx-1-cpa-10.1177_07067437211055412 - Supplemental material for Diagnosed Incidence of Non-Affective Psychotic Disorders Amongst Adolescents in British Columbia and Sociodemographic Risk Factors: A Retrospective Cohort Study [file sj-docx-1-cpa-10.1177_07067437211055412.docx]

**Online Supplementary Material: Sensitivity Analyses with Validated Algorithm to Detect Chronic Psychotic Illness**

We conducted sensitivity analyses using an algorithm validated in Ontario to detect chronic psychotic illness^33^ to ascertain cases: at least one hospitalization or two outpatient visits with a primary diagnosis for non-affective psychotic disorder (ICD-9 295, 298; ICD-10 F20, F25, F29).

**RESULTS**

**Diagnosed incidence based on validated algorithm to detect chronic psychotic illness**

We identified 1,153 cases (438 female and 715 male) of non-affective psychotic disorder onset between the ages of 13 and 19. Cumulative incidence of non-affective psychotic disorder by age 19 was .49% [95% CI: .44, .54] among females and .75% [95% CI: .70, .81] among males. Incidence rate over the entire study period was 80.8 per 100,000 person-years [95% CI: 73.6, 88.7] for females and 124.1 per 100,000 person-years [95% CI: 115.4, 133.6] for males.

**Cox proportional hazards model results based on validated algorithm to detect chronic psychotic illness**

Table S1 shows the Cox proportional hazards regression results based on the validated algorithm for detecting chronic psychotic illness by sex with 95% confidence intervals. Low family income was associated with significantly elevated risk of diagnosis (aHR females=1.99[95% CI: 1.63, 2.43] and aHR males=1.77[95% CI: 1.51, 2.07]). Among females, the hazard ratio comparing the lowest and highest neighbourhood income quintiles was statistically significant in the unadjusted model only (HR=1.40[95% CI: 1.05, 1.87]). Among males, the lowest two quintiles (aHR=1.39[95% CI: 1.08, 1.79] and aHR=1.34[95% CI: 1.04, 1.72]) and those with missing neighbourhood income information (aHR=2.35[95% CI: 1.31, 4.20]) exhibited significantly elevated risk compared to the highest quintile. Children of immigrants had significantly lower risk of diagnosis compared to children of non-migrants (aHR females=0.65[95% CI: 0.50, 0.83] and aHR males=0.66[95% CI: 0.54, 0.80]). Children of refugees had lower risk of diagnosis compared to children of non-migrants (aHR females=0.62[95% CI: 0.35, 1.08] and aHR males=0.71[95% CI: 0.47, 1.08); however, these effect estimates were not statistically significant in any models. Finally, elevated risk of diagnosis was observed among individuals with a parent with a health contact for an anxiety/mood/substance disorder (aHR females=1.56[95% CI: 1.28, 1.89] and aHR males=1.66[95% CI: 1.43, 1.93]), and for a non-affective psychotic disorder (aHR females=4.22[95% CI: 2.62, 6.77] and aHR males=4.29[95% CI: 2.94, 6.26]) and among those born in 1996-98 (aHR females=1.46[95% CI: 1.16, 1.85] and aHR males=1.33[95% CI: 1.11, 1.61]) compared to those born in 1990-92. Those born in 1993-95 had elevated risk of diagnosis compared to those born in 1990-92 but this effect was not statistically significant (aHR females=1.16[95% CI: 0.92, 1.45] and aHR males=1.19[95% CI: 1.00, 1.42]).

Table S1. Cox proportional hazards regression results using validated algorithm to detect chronic psychotic illness

|  | Females (n=93,784) | | Males (n= 99,616) | |
| --- | --- | --- | --- | --- |
|  | HR [95% CI] | aHR [95% CI] | HR [95% CI] | aHR [95% CI] |
| Low Family Income |  |  |  |  |
| No | Reference | Reference | Reference | Reference |
| Yes | 2.00 [1.66, 2.42] | 1.99 [1.63, 2.43] | 1.84 [1.58, 2.13] | 1.77 [1.51, 2.07] |
| Missing | 0.69 [0.29, 1.68] | 0.81 [0.34,1.98] | 1.00 [0.57, 1.78] | 1.16 [0.65, 2.06] |
| Neighbourhood income quintile | |  |  |  |
| 5 (highest) | Reference | Reference | Reference | Reference |
| 4 | 0.75 [0.54, 1.04] | 0.72 [0.52,1.01] | 1.12 [0.86, 1.45] | 1.07 [0.82, 1.39] |
| 3 | 1.05 [0.78, 1.42] | 0.99 [0.73, 1.34] | 1.17 [0.90, 1.52] | 1.10 [0.85, 1.43] |
| 2 | 0.97 [0.71, 1.32] | 0.86 [0.63, 1.17] | 1.48 [1.16, 1.89] | 1.34 [1.04, 1.72] |
| 1 (lowest) | 1.40 [1.05, 1.87] | 1.15 [0.85, 1.55] | 1.62 [1.26, 2.07] | 1.39 [1.08, 1.79] |
| Missing | 0.82 [0.26, 2.60] | 0.64 [0.50, 2.03] | 2.92 [1.64, 5.20] | 2.35 [1.31, 4.20] |
| Family migration background | |  |  |  |
| Non-migrant | Reference | Reference | Reference | Reference |
| Immigrant | 0.70 [0.55, 0.90] | 0.65 [0.50, 0.83] | 0.70 [0.58, 0.85] | 0.66 [0.54, 0.80] |
| Refugee | 0.81 [0.47, 1.41] | 0.62, [0.35, 1.08] | 0.91 [0.60, 1.38] | 0.71 [0.47, 1.08] |
| Parent MH contact |  |  |  |  |
| None | Reference | Reference | Reference | Reference |
| Anxiety/mood/substance | 1.65 [1.36, 1.99] | 1.56 [1.28, 1.89] | 1.74 [1.50, 2.02] | 1.66 [1.43, 1.93] |
| Non-affective psychotic | 5.38 [3.36, 8.61] | 4.22 [2.62, 6.77] | 5.25[3.61, 7.64] | 4.29 [2.94, 6.26] |
| Birth year |  |  |  |  |
| 1990 to 92 | Reference | Reference | Reference | Reference |
| 1993 to 95 | 1.16 [0.92, 1.45] | 1.16 [0.92, 1.46] | 1.19 [0.98, 1.42] | 1.19 [1.00, 1.42] |
| 1996 to 98 | 1.42 [1.13, 1.80] | 1.46 [1.16, 1.85] | 1.29 [1.07, 1.55] | 1.33 [1.11, 1.61] |
| *Note*. HR=unadjusted hazard ratio; aHR=adjusted hazard ratio. aHR are adjusted for all other explanatory variables. | | | | |
